# Identification of Novel Candidate Genes for Early-Onset Colorectal Cancer Susceptibility

**DOI:** 10.1371/journal.pgen.1005880

**Published:** 2016-02-22

**Authors:** Richarda M. de Voer, Marc-Manuel Hahn, Robbert D. A. Weren, Arjen R. Mensenkamp, Christian Gilissen, Wendy A. van Zelst-Stams, Liesbeth Spruijt, C. Marleen Kets, Junxiao Zhang, Hanka Venselaar, Lilian Vreede, Nil Schubert, Marloes Tychon, Ronny Derks, Hans K. Schackert, Ad Geurts van Kessel, Nicoline Hoogerbrugge, Marjolijn J. L. Ligtenberg, Roland P. Kuiper

**Affiliations:** 1 Department of Human Genetics, Radboud University Medical Center, Nijmegen, The Netherlands; 2 Center for Molecular and Biomolecular Informatics, Radboud Institute for Molecular Life Sciences, Radboud University Medical Center, Nijmegen, The Netherlands; 3 Abteilung Chirurgische Forschung, Technische Universität Dresden, Dresden, Germany; 4 Department of Pathology, Radboud University Medical Center, Nijmegen, The Netherlands; Brigham and Women's Hospital, UNITED STATES

## Abstract

Approximately 25–30% of colorectal cancer (CRC) cases are expected to result from a genetic predisposition, but in only 5–10% of these cases highly penetrant germline mutations are found. The remaining CRC heritability is still unexplained, and may be caused by a hitherto-undefined set of rare variants with a moderately penetrant risk. Here we aimed to identify novel risk factors for early-onset CRC using whole-exome sequencing, which was performed on a cohort of CRC individuals (*n =* 55) with a disease onset before 45 years of age. We searched for genes that were recurrently affected by rare variants (minor allele frequency ≤0.001) with potentially damaging effects and, subsequently, re-sequenced the candidate genes in a replication cohort of 174 early-onset or familial CRC individuals. Two functionally relevant genes with low frequency variants with potentially damaging effects, *PTPN12* and *LRP6*, were found in at least three individuals. The protein tyrosine phosphatase PTP-PEST, encoded by *PTPN12*, is a regulator of cell motility and LRP6 is a component of the WNT-FZD-LRP5-LRP6 complex that triggers WNT signaling. All variants in *LRP6* were identified in individuals with an extremely early-onset of the disease (≤30 years of age), and two of the three variants showed increased WNT signaling activity *in vitro*. In conclusion, we present *PTPN12* and *LRP6* as novel candidates contributing to the heterogeneous susceptibility to CRC.

## Author Summary

Onset of colorectal cancer (CRC) at an early age in life is suggestive of a hereditary predisposition. However, a large proportion of the heritability of early-onset CRC is still unexplained. We aimed to identify novel genes that may confer CRC susceptibility by whole-exome sequencing of 55 individuals that were diagnosed with this disease before the age of 45 years. To identify candidate causative variants, we applied a strategy in which we searched for genes with multiple rare (minor allele frequency ≤0.001) variants in cases but not in controls with a potentially damaging effect, and with a functional link to (colorectal) cancer development. Based on targeted re-sequencing and functional analyses we identified *LRP6* and *PTPN12* as novel candidate genes for CRC susceptibility. A validation of these genes in larger independent case and control cohorts is needed to establish the precise role of these genes in the susceptibility to develop CRC.

## Introduction

Colorectal cancer (CRC) is a heterogeneous disease with an estimated heritable component of 25–30%. About 5–10% of CRC cases are currently explained by germline mutations in genes that predispose to Mendelian cancer syndromes, such as Lynch syndrome, Familial Adomatous Polyposis, Peutz-Jeghers syndrome, Juvenile Polyposis syndrome, *MUTYH*-associated polyposis, *NTHL1*-associated polyposis, and Polymerase Proofreading-associated Polyposis syndrome.[1–5] Typically, these CRC syndromes are marked by a strong family history, a high-penetrance of the disease, and the development of multiple tumors at an early age.[1] An additional 10% of the heritability of CRC may be explained by a growing list of common, low penetrant risk factors.[5–7] In spite of this, a large part of the heritability of CRC still remains unexplained and may well result from the presence of rare variants with a moderate risk.[8]

Moderately penetrant risk factors do not necessarily result in dominant traits, since their phenotypic effects may rely on an interplay with other genetic and/or environmental factors.[2] However, these genetic factors may play a role in individuals with an early-onset of the disease, one of the hallmarks of hereditary cancer.[9] In addition, unexplained early-onset CRC cases differ from late-onset sporadic CRC cases based on their distinct clinical, molecular, and pathological etiology.[10]

Here, we used whole-exome sequencing to identify novel moderately penetrant CRC risk factors. Since we expect such factors to follow non-Mendelian inheritance patterns, we focused specifically on individuals with an early onset of the disease, which frequently manifests in the absence of a clear family history. We selected recurrent rare variants in genes with a low burden of comparable variants in controls, and identified *PTPN12* and *LRP6* as novel candidate risk factors for early-onset CRC.

## Results

### Whole-exome sequencing of early-onset CRC cases

**Fig 1:**
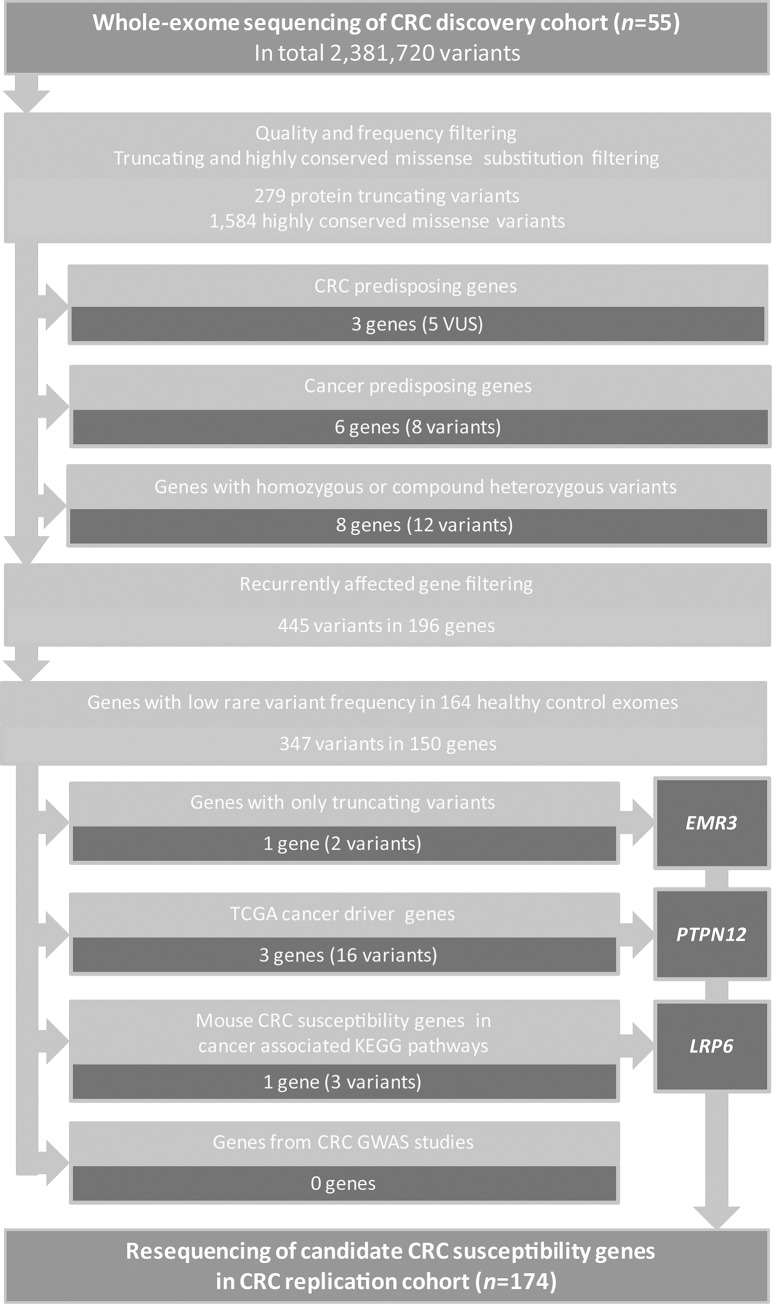
Study design, variant filtering and candidate gene prioritization. Whole-exome sequencing was performed on germline DNA of 55 early-onset CRC cases. The exome data were first filtered for quality and frequency, followed by filtering for protein truncating and highly conserved missense variants. Next, we removed all known loss-of-function-tolerant genes from this list and searched for known and novel CRC predisposing gene variants.[12,13,57] An additional filtering was applied to identify genes that were affected by two or more potentially pathogenic variants and to remove genes that are frequently affected by protein-truncating or highly conserved missense variants in healthy controls. The remaining set of recurrent variants was filtered for (i) genes recurrently affected by protein truncating variants; (ii) cancer driver genes in CRC [23]; (iii) genes identified as CRC susceptibility genes in mice [29,30] and involved in cancer-related KEGG pathways [hsa04310 (WNT signaling), hsa04350 (TGF-beta signaling), hsa03430 (mismatch repair), hsa03410 (base excision repair), hsa03420 (nucleotide excision repair), map03450 (non-homologous end-joining), hsa03460 (Fanconi anemia), and hsa05200 (pathways in cancer)] (iv) genes identified in CRC GWAS studies [5–7,32]. Genes that remained after these filter steps were selected for re-sequencing in a replication cohort of 174 CRC cases. CRC: colorectal cancer; VUS: variant of unknown significance.

To identify rare genetic variants involved in CRC susceptibility, we performed whole-exome sequencing on germline DNA from 55 CRC cases, diagnosed at age 45 years or younger. The average age at diagnosis was 35 years [range: 23–45] and 22 cases (40%) had a positive family history for cancer (Table 1 and S1 Table). The average coverage per target region was 76x [range: 46-127x] with on average 43,124 identified variants [range: 29,040–49,929] per exome (detailed metrics per exome are listed in S2 Table). In total 2,381,720 variants were identified. After quality assessment, common variants were excluded, which were defined as variants that are present in our in-house database with 2,037 exomes from mostly Western-European ancestry or the Exome variant server (EVS) with minor allele frequencies (MAF) >0.001.[11] Subsequently, we selected variants that result in protein truncation (i.e. frameshift or nonsense mutations and variants at canonical splice sites) and missense substitutions at highly conserved positions (non-synonymous variants with a PhyloP score ≥3; see Materials and Methods for a detailed description). We identified on average 34 [range: 10–89] rare variants per sample, including on average 5 [range: 1–10] protein truncating variants and 29 [range: 8–83] missense variants. In total, 279 protein truncating and 1,584 missense variants were identified (S3 Table). These potentially pathogenic variants were subjected to a subsequent filtering strategy to identify known and novel CRC predisposing gene variants (Fig 1).

**Table 1:** Clinical characteristics of the discovery and replication cohorts with mismatch-repair proficient colorectal cancer.

Characteristic		Exome cohort (%) *N* = 55	Dutch replication cohort (%) *N* = 82	German replication cohort (%) *N* = 92
**Average age of onset (SD)**		35 (4)	40.4 (4.5)	37.7 (5.6)
**Average age**				
	≤25 yr	2 (4)	1 (1)	1 (1)
	25–30 yr	8 (15)	2 (2)	12 (13)
	31–35 yr	17 (31)	10 (12)	16 (17)
	36–40 yr	26 (47)	21 (26)	27 (29)
	40–45 yr	2 (4)	46 (56)	35 (38)
	45–50 yr	-	2 (2)	1 (1)
**Family history for cancer**				
	None	27 (49)	23 (28)	4 (4)
	≥one first degree relative	10 (18)	33 (40)	37 (3)
	≥one second degree relative	10 (18)	13 (16)	20 (22)
	≥one third degree relatives	2 (4)	1 (1)	1 (1)
	Unknown	6 (11)	12 (15)	30 (33)
**Tumor location**				
	Right colon	8 (15)	7 (9)	15 (16)
	Transverse colon	4 (7)	8 (10)	9 (10)
	Left colon	13 (24)	13 (16)	25 (27)
	Rectum	25 (46)	32 (39)	33 (36)
	Unknown	6 (11)	19 (23)	10 (11)

### Variants in (colorectal) cancer predisposing genes

Within the list of potentially pathogenic variants, we analyzed whether these variants were present in any of the known CRC predisposing genes, i.e, mismatch repair (MMR) genes, *APC*, *POLD1*, *POLE*, *SMAD4*, *BMPR1A*, *MUTYH*, *NTHL1*. In three individuals, variants in the MMR genes *MSH2*, *MSH6* (three variants) and *PMS2* were identified (S4 Table), but based on microsatellite instability or immunohistochemistry analyses none of these variants resulted in MMR deficiency. Therefore, these variants were allocated to be of unknown significance.

Next, we focused on variants in genes known to predispose to other types of cancer or to cause cancer syndromes when present in a biallelic state.[12,13] We identified eight variants in six different genes (S5 Table). Two of these genes, *BLM* and *ATM*, were found to harbor rare variants in two patients. *BLM* encodes a *RECQL*-DNA helicase and is involved in the recessive Bloom syndrome, which predisposes to CRC. Although current literature on the risk of CRC development in carriers of pathogenic *BLM* alleles is contradictory [14–16], we recently found by targeted re-sequencing of a validation cohort that monoallelic pathogenic *BLM* variants were enriched in these cases.[17] ATM is involved in double-strand break repair and mutations in its encoding gene are associated with ataxia telangiectasia, another autosomal recessive disorder. Somatic nonsense mutations in *ATM* occur frequently in CRC, but monoallelic germline mutations have thus far only been shown to increase the risk for gastric, breast and pancreatic cancers, and mostly when they result in truncated proteins.[18–20] Potentially pathogenic missense variants in *ATM* were also found twice in a cohort of 164 healthy individuals (Control dataset 1; see Materials and Methods and below) and we, therefore, conclude that *ATM* is not enriched in our cohort of CRC cases. The remaining four variants represent highly conserved missense or protein truncating variants that occurred in four different genes: *BRIP1*, *EGFR*, *ERCC3* and *WRN* (S5 Table).

### Identification of novel CRC susceptibility genes

To identify novel candidate CRC susceptibility genes, we first focused on genes with biallelic mutations (recessive inheritance pattern). Therefore, we analysed our list of potentially pathogenic variants for the presence of autosomal homozygous (≥95% variant reads) or compound heterozygous variants and, subsequently, performed Sanger validation. We identified biallelic variants in eight individuals, all affecting different genes (Fig 1, S6 Table). However, these genes were either found to be be linked to other phenotypes, to contain many rare variants in the general population, or to have a function that remains to be elucidated. Therefore, these variants were excluded from further analysis.

Subsequently, we filtered our list of potentially pathogenic variants for genes with at least two variants in unrelated individuals (see Materials and Methods for details). In total, we identified 445 rare potentially pathogenic variants in 196 recurrently affected genes. Some of these genes contain many rare variants that occur in the general population and are, thus, unlikely to provoke an increased risk for CRC. Therefore, we selected an exome sequencing data set from healthy individuals (Control dataset 1; *n* = 164) that were subjected to the same procedure as our CRC cohort and were found to be comparable based on genotyping accuracy and population (S1 Fig; see Materials and Methods for a detailed description). This ‘Control dataset 1’ was used to remove genes from our list that were not significantly enriched in the CRC cohort after a χ^2^ goodness of fit test (S7 Table; Materials and Methods). The remaining variants, i.e., 347 variants in 150 genes, were subjected to a four-step prioritization as outlined below and depicted in Fig 1.

First, we investigated whether genes were recurrently affected by rare protein truncating variants. Only one gene, *EMR3*, was found to fulfill this criterion with two splice site mutations (Table 2), each predicted to result in skipping of an exon. *EMR3* belongs to the EGF-TM7 protein family of G-protein coupled receptors and is specifically expressed in granulocytes and data from ‘The Human Protein Atlas’ suggest that EMR3 is not or only very lowly expressed in colonic tissue.[21,22] *EMR3* has been suggested to function as a mediator of invasive phenotypic variation in glioblastoma.[23]

**Table 2:** Variants in candidate CRC susceptibility genes identified by whole-exome sequencing and targeted re-sequencing.

Gene	Sample Names	Transcript	Variant			Pathogenicity				Variant Frequency (*n*; MAF)					
		Refseq. accession	Nucleotide (cDNA)	Amino acid (protein)	dbSNP	PhyloP	SIFT	Poly Phen-2	A-AVGD^a^	Discovery cohort (*n* = 55)	In-house DB (*n* = 2,037)	EVS (*n* = 6,503)	Control cohort 2 (*n* = 2,329)	ExAC (*n* = 60,706)	Replication cohort (*n* = 174)
*EMR3*	P025	NM_032571	c.882+1G>A	p.?	N/A	2.427	N/A	N/A	N/A	1 (0.009091)	0	1 (0.0000769)	0	11 (0.0000914)	0
	P002		c.1249-2A>C	p.?	N/A	3.787	N/A	N/A	N/A	1 (0.009091)	0	0	1 (0.0002147)	0	0
*PTPN12*	P014	NM_002835	c.1565G>T	p.R522M	N/A	4.531	Del.	Prob.dam.	C25	1 (0.009091)	0	0	2 (0.0004294)	0	0
	P045 & P054		c.2051C>T	p.S684L	rs201001953	3.958	Del.	Prob.dam.	C0	2 (0.018182)	0	3 (0.0002307)	3 (0.0006441)	111 (0.000914)	0
	RC204		c.314C>T	p.A105V	N/A	4.24	Del.	Prob.dam.	C65	0	0	0	0	0	1
*LRP6*	P002	NM_002336	c.716C>A	p.W239L	N/A	5.103	Del.	Prob.dam.	C65	1 (0.009091)	0	0	0	0	0
	P001		c.2366T>C	p.N789S	N/A	6.244	Del.	Benign	C55	1 (0.009091)	0	0	0	0	0
	P008		c.2599A>G	p.T867A	rs141458215	5.13	Del	Benign	C55	1^b^ (0.009091)	0	0	3 (0.0006441)	14 (0.000115)	0

^a^ Align-GVGD scores vary from C0 (benign) to C65 (damaging).

^b^ Did not pass the original quality settings.

N/A, not applicable; ND, not done; Del., deleterious; Prob.dam., probably damaging; Pos.dam., possibly damaging; EVS, exome variant server [11]; ExAC, Exome Aggregation Consortium [33].

**Fig 2:**
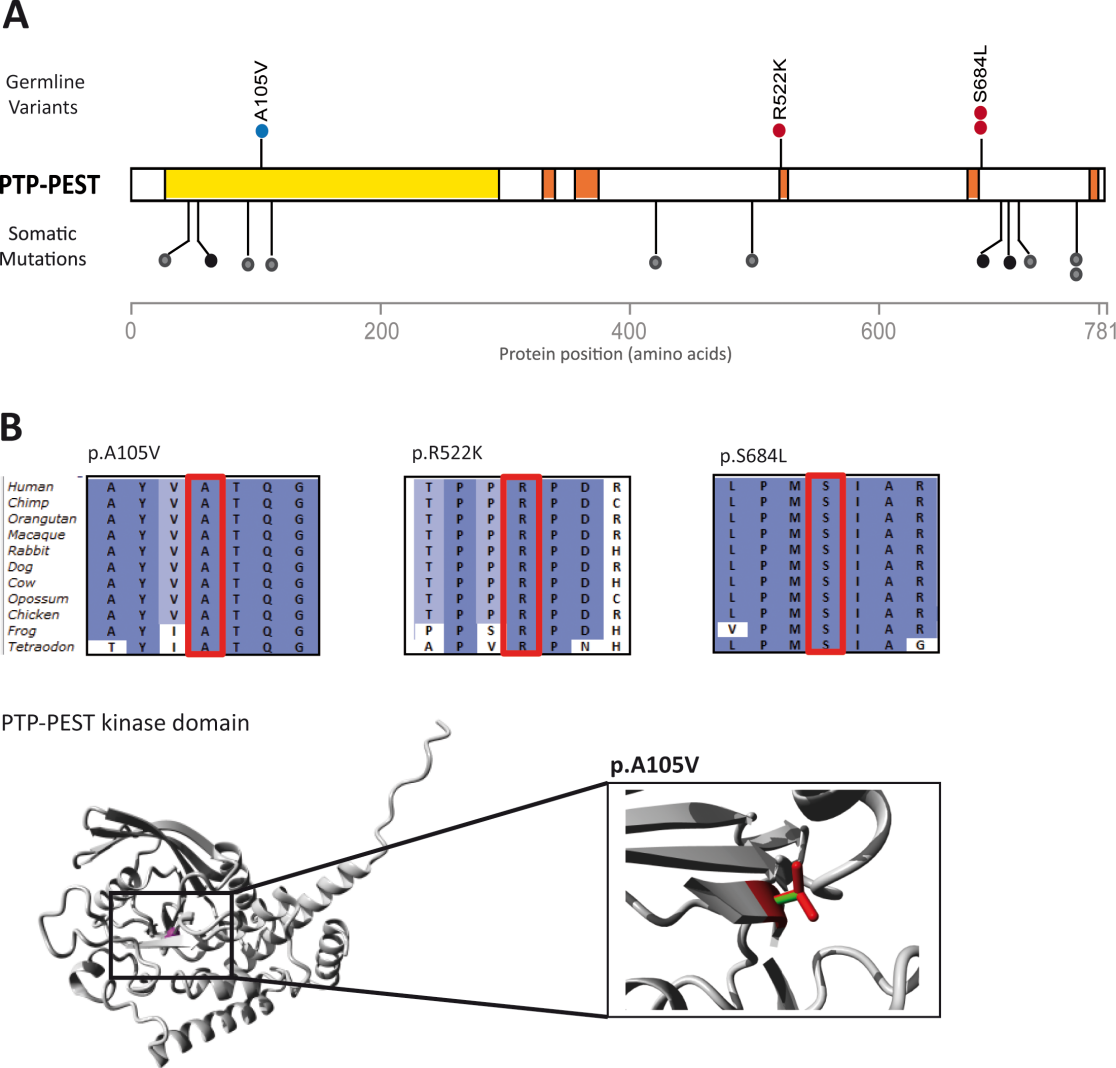
Rare variants in *PTPN12* encoding PTP-PEST in four cases. (A) Distribution of missense variants identified in the CRC discovery cohort (red dots) and CRC replication cohort (blue dots) in PTP-PEST. Somatic *PTPN12* mutations identified in colorectal tumors [24] are indicated with black (missense) and green (protein truncating) dots. The kinase domain is shown in yellow and the proline-rich-regions are shown in orange. (B) Amino acid conservation of the three regions with missense variants (indicated with the red box) among 11 species, and the 3D protein structure of the kinase domain with the p.A105V variant. The close up shows the structural difference between the mutant (red) and wild-type (green) residue. The mutant residue at position 105 is bigger and may cause bumps during protein folding. The mutant residue at position 522 is smaller, which can result in loss of interactions. The mutant residue at position 684 is more hydrophobic than the wild-type residue, this may disturb correct protein folding.

Second, we selected variants in genes that were identified as potential CRC driver genes (S8 Table).[24] We identified 16 variants in three genes: *MSH6* (3 variants), *TTN* (10 variants) and *PTPN12* (3 variants). The tumors of the carriers of the *MSH6* variants do not reveal MMR deficiency and *TTN* is known to harbor an excess of rare private variants, is mainly expressed in cardiac and skeletal muscle, and is associated with cardiomyopathies (MIM: 188840). Therefore, we investigated the three rare variants in *PTPN12* in more detail (Table 2). *PTPN12* encodes the protein tyrosine phosphatase PTP-PEST that functions as a suppressor of epithelial cell motility in CRC cells and has been linked to breast cancer development.[25–27] The identified variants p.S684L (2 cases, subjects P045 and P054) and p.R522K (1 case, subject P014) were found to be located adjacent to the proline-rich regions of PTP-PEST (Fig 2), which have been shown to interfere with the kinase activity of PTP-PEST.[25–28] A data search in ‘The Human Protein Atlas’ revealed that PTPN12 is expressed at high levels in colonic tissue.[22] Somatic mutations that have been reported in *PTPN12* were found to result in truncations of the protein or in amino acid substitutions (Fig 2A).[24]

**Fig 3:**
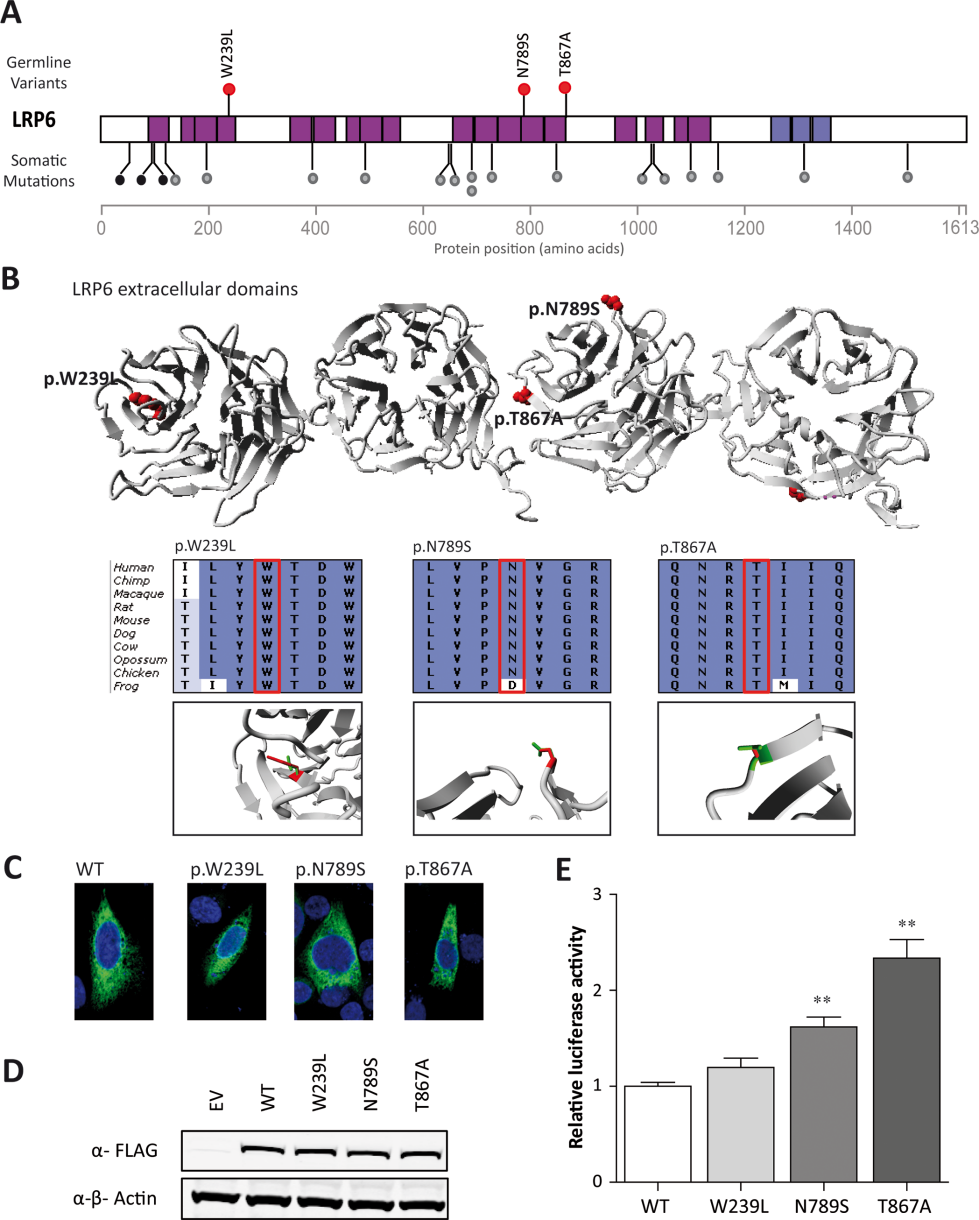
Rare variants in *LRP6* in three cases. (A) Distribution of missense *LRP6* variants identified in the CRC discovery cohort (red dots). Somatic *LRP6* mutations identified in colorectal tumors [24] are indicated with grey (missense) and black (protein truncating) dots. Structural domains include the β-propeller domains that are used to form the receptor complex (pink bars), and the transmembrane domain (purple). (B) 3D protein structure of the β-propeller domains of LRP6 with the positions of the identified missense variants in red. Insets show conservation of the region in which the missense variants (indicated with the red box) are located with, underneath, close ups of the local 3D protein structure with mutant (red) and wild-type (green) residues. The mutant residue at position 239 is predicted to disturb the protein structure (project HOPE; http://www.cmbi.ru.nl/hope/). The mutant residue at position 789 is much smaller than the wild-type residue and may disturb the binding of Dickkopf-1. Residue 867 is located on the surface of the protein and the mutant residues are not expected to disturb protein structure, but may influence protein binding. (C) Immunofluorescence analyses of LRP6 wild-type and mutant proteins showing similar subcellular localizations. (D) LRP6 protein expression levels normalized to β-actin are similar between wild-type and mutant LRP6. (E) TOPflash analyses of wild-type and mutant LRP6 to determine their effects on the WNT signaling pathway. Luciferase activity was normalized to control and wild-type constructs. Both p.N789S and p.T867A mutants reveal a significant increase in activation compared to the wild-type LRP6 protein. Experiments were performed three times in triplicate. **P <0.001; error bars represent the standard error of the mean.

Third, we filtered for variants in genes that were identified as mouse CRC susceptibility genes in transposon-based studies [29,30]. We identified 18 variants in eight such genes (S9 Table). Since most of these genes did not exhibit a clear link to cancer development, we filtered our list for genes for those that are described in KEGG pathways implicated in CRC development. This resulted in one remaining candidate, namely *LRP6*. In three CRC patients diagnosed before the age of 30 highly conserved *LRP6* missense variants with a predicted pathogenicity, p.W239L (subject P002), p.N789S (subject P001) and p.T867A (subject P008), were identified (Table 2). *LRP6* encodes a receptor of the WNT-FZD-LRP5-LRP6 complex that triggers β-catenin signaling. The LRP6 protein consists of four β-propeller domains, a transmembrane domain, and a cytoplasmic domain. All variants were located in β-propeller domains (Fig 3A and 3B), which are involved in the binding of WNT ligands and antagonists, such as WNT3a and Dickkopf-1, respectively.[31] Data from ‘The Human Protein Atlas’ revealed that LRP6 is expressed at high levels in colonic tissue.[22] Interestingly, *LRP6* has also been found to be affected by somatic mutations in colorectal adenocarcinomas,[24] in which most of these mutations appear to occur within in the β-propeller domains (Fig 3A), thus supporting a role of missense variants in this gene in CRC development.

Fourth, we examined whether any of the genes that were previously identified in familial CRC genome-wide association studies (GWAS)[32] were present in our list of genes recurrently affected by rare variants, but none were identified.

### Frequency of identified variants in control populations

We analyzed the frequency of the potentially pathogenic variants that we identified in the *EMR3*, *PTPN12* and *LRP6* genes in an additional cohort of locally sequenced control individuals without any suspicion for hereditary cancer, and in data from the Exome Aggregation Consortium.[33] The local control individuals were sequenced at a high coverage and exhibited sufficient read depth for our three candidate genes (Control dataset 2; *n =* 2,329; S2 Fig and Materials and Methods for a detailed description). All variants that we identified in *EMR3*, *PTPN12* and *LRP6* were either absent or present at an extremely low frequency in both these data sets (MAF of ≤0.001; Table 1). Next, we determined the number of variants in these three genes with a similar predicted effect in ‘Control dataset 2’. This analysis revealed that protein-truncating variants in *EMR3* and missense variants with a PhyloP ≥3.0 in *PTPN12* and *LRP6* are indeed enriched in our cohort of CRC patients, which remained significant after correcting for multiple testing for three genes. After correcting for exome-wide multiple testing, none of the genes were significantly enriched (S10 Table).

### Resequencing of novel candidate CRC susceptibility genes

Next, we performed targeted resequencing of the novel candidate CRC susceptibility genes *EMR3*, *PTPN12* and *LRP6* using an Ion Ampliseq custom panel (Life technologies, see Materials and Methods for details) and a replication cohort of 174 CRC cases (Table 1). With an average read-depth of 530-fold per targeted region [range: 21–1,852] per sample, one additional rare potentially damaging variant in the kinase domain of the PTPN12 protein was identified (p.A105V, subject RC204, age 45 years) and confirmed by Sanger sequencing (Table 2 and Fig 2). This is not a significant increase compared to ‘Control dataset 2’ (S10 Table). No additional variants in *EMR3* and *LRP6* were identified.

### Missense variants in *LRP6* activate WNT signaling pathway

The three *LRP6* variants identified were all encountered in individuals with a very early-onset of the disease: 23, 24 and 29 years of age. To assess whether these *LRP6* variants may have an effect on the WNT signaling pathway, we performed TOPflash assays with LRP6 wild-type (WT) and mutant proteins. Immunofluoresence and Western blot analyses revealed similar localization patterns and equal expression levels of the WT and mutant LRP6 proteins (Fig 3C and 3D). Overexpression of the mutant LRP6 proteins (p.N789S and p.T867A) induced 1.6-fold (*P* = 0.0003) and 2.4-fold (*P* <0.0001) increases in WNT signaling activity in the TOPflash assay compared to the LRP6 WT protein, respectively (Fig 3E).

### Cosegregation analysis

Based on the availability of suitable material, a cosegregation analysis could only be performed for subject P002 (p.W239L; age 23 years). P002 did not have a clear family history of CRC, but her mother, two sisters and an aunt developed breast cancer. We found that the mother also carried the p.W239L variant. As we were unable to perform a complete cosegregation analysis in this family, or in any of the other *PTPN12* or *LRP6* families, it remains to be established to what extend carriers of variants in these genes tend to develop cancer at a young age.

## Discussion

In this study we have performed whole-exome sequencing on germline DNA from 55 mismatch repair-proficient early-onset CRC cases and identified multiple potentially damaging variants in two colon-expressed genes: *PTPN12* and *LRP6*. The germline variants identified in these genes appeared to affect highly conserved amino acids and were absent, or present at extremely low frequencies, in control populations. Targeted re-sequencing of a replication cohort of 174 individuals with early-onset CRC revealed one additional variant in *PTPN12*. We also found that two of the three missense variants in LRP6 can activate the WNT pathway *in vitro*. Based on these results, we propose that *PTPN12* and *LRP6* serve as novel moderately penetrant CRC susceptibility genes.

Within our discovery cohort, we focused on genes that were recurrently affected by rare potentially damaging variants. Rare variants represent the vast majority of normal variation in the human genome and they are unequally distributed between genes and between biological pathways,[34–37] illustrating that a comparison with exome sequence data from a geographically matched control cohort is crucial. We, therefore, selected only variants with a MAF ≤0.001 in locally sequenced control individuals, and applied the entire analysis pipeline to a dataset of 164 healthy controls that were sequenced using the same exome enrichment and sequencing procedures. This approach allowed us to reduce the risk of selecting locally common benign variants, to prevent the selection of false-positives by technical errors, and to focus only on genes that are protected from gathering pathogenic mutations in the normal population.[38] Furthermore, we analyzed an additional, more recent, high quality exome sequencing dataset from local individuals to confirm that our identified variants were indeed extremely rare.

One of the candidate genes, *PTPN12*, encodes the widely expressed cytoplasmic protein tyrosine phosphatase PTP-PEST, which regulates epithelial cell adhesion and migration.[25,39–41] In colon carcinoma cells, PTP-PEST has been shown to control cell motility and adherence junction assembly by regulating the intracellular localization of p120 catenin and, consequently, its interaction with E-cadherin.[27] Therefore, PTP-PEST may play a role in the tightly controlled migration of epithelial cells of the colonic crypts. The highly conserved variants identified in our study may result in diminished phosphatase activity, as has been demonstrated for comparable missense variants in the same domains,[26,28] resulting in aberrant crypt formation and possibly invasion.

*LRP6* is a core-component of the WNT-FZD-LRP5-LRP6 receptor complex of the WNT signaling cascade, which is commonly activated in colon cancer.[42] Previously, this gene was also identified in a screen for mouse CRC susceptibility loci.[30] Remarkably, we found that the rare *LRP6* variants were present in three of the youngest diagnosed individuals in our cohorts (age 23, 24 and 29). Furthermore, two of the identified missense variants in *LRP6* were found to be located at positions of the protein that interact with the WNT antagonist Dickkopf-1 [43] and to result in significantly increased activations of the WNT pathway in *in vitro* TOPflash reporter assays. This finding is intriguing, as it was recently shown that missense variants in *LRP6* can abrogate activation of the WNT pathway.[44]

Despite the fact that *EMR3* is recurrently affected by rare protein truncating variants in our discovery cohort, we do not consider *EMR3* a strong candidate gene for CRC susceptibility, since the protein is not or only very lowly expressed in tissue from colon and rectum, and we could not establish a functional link to CRC development.[21–23]

Several recent whole-exome sequencing studies have revealed novel candidate CRC susceptibility genes and variants,[45–51] but the concordance between these findings, including our own, is as yet limited, and statistical evidence for these correlations in case-control studies was not obtained. This limited concordance may be due to different patient inclusion criteria used, ranging from the selection of predominantly familial cases to individuals with isolated CRC at young age. In addition, it cannot be excluded that non-genetic (environmental) risk factors may have played a role in the development of CRC at a younger age in individual cases. Clearly, the genetic heterogeneity of CRC susceptibility may also, at least partly, explain the limited concordance between studies, and points out that statistical validation required very large case and control cohorts. Optimal phenotypic pre-selection, including genetic or histologic abnormalities in the tumor, or even the presence of additional congenital features as we recently identified in individuals with *BUB1* and *BUB3* abnormalities,[52] may limit this heterogeneity. Furthermore, variant selection varies between studies. So far, most studies have put a primary focus on high-penetrance germline mutations by showing cosegregation within families and second-hit mutations in the tumor,[45–50] but this approach may not be valid for predisposing variants with a moderate penetrance.

The inclusion of missense variants as potential candidates is challenging, because their effect is difficult to predict, and can vary widely between variants in a particular gene. Nevertheless, the putative relevance of missense variants in functionally important domains should not be underestimated, as was recently illustrated by the discovery of missense *POLE* and *POLD1* variants.[4] Functionally important domains are conserved both at the nucleic acid and the amino acid level. Currently, there is no consensus on the best strategy to discriminate pathogenic missense variants from benign missense variants, which poses a challenge to this type of cohort studies. We have used PhyloP scores as the first prioritization cut-off in the pathogenicity assessments, as it was previously shown that pathogenic variants have higher scores than benign variants,[53] This prioritization step was followed by other *in silico* steps to predict pathogenicity based on the biophysical amino acid characteristics and multiple protein sequence alignments. Finally, essential evidence for the role of damaging variants in novel genes may results from the presence of these variants in independent families, but their frequency is expected to be low. Therefore, large replication series are needed that encompass comparable cases to replicate the findings. Additionally, more and better candidates are likely to be identified when datasets from different studies are merged and re-analyzed. We did, however, identify several rare variants in single individuals in candidate CRC susceptibility genes previously reported by others (S11 Table). The same may, in reverse, turn out to be true for *PTPN12* and *LRP6*.

In conclusion, we and others have shown by whole-exome sequencing that the genetic susceptibility to CRC is heterogeneous. Our findings are in line with a polygenic model of CRC susceptibility in which multiple risk factors in known and novel CRC pathways may contribute to additive risks.[54] Future studies, such as functional assessments of the candidate genes and replications in larger CRC and control cohorts, are needed to firmly establish the role of *PTPN12* and *LRP6* in CRC susceptibility.

## Materials and Methods

### Study samples

The discovery cohort used consists of 55 non-polyposis DNA mismatch repair (MMR)-proficient CRC cases, diagnosed at ≤45 years of age, which were referred to the Radboud university medical center, Nijmegen, the Netherlands (Table 1 and S1 Table).[12,52] The replication cohort used consists of 174 MMR-proficient CRC cases from both Nijmegen, The Netherlands, and Dresden, Germany, that fulfilled one of the following two criteria: (i) diagnosed with CRC ≤40 years of age (*n* = 90) regardless of family history, or (ii) diagnosed with CRC ≤50 years of age with at least two first-degree relatives with CRC (n = 84). All individuals provided written informed consent. The study was approved by the CMO (study number 2009/256), Arnhem and Nijmegen, the Netherlands.

### Whole-exome sequencing

Whole-exome sequencing was performed on genomic DNA extracted from peripheral blood cells of cases included in the CRC discovery cohort with SOLiD4 (*n =* 10) and 5500xl (*n =* 45) sequencing platforms (Life technologies, Carlsbad, CA, USA) using the SOLiD-optimized SureSelect Human All Exon kit V1 (30Mb set, *n =* 1), V2 (50Mb set, *n =* 44) and V4 (50Mb set, *n =* 10; Agilent Technologies, Santa Clara, CA, USA) as described previously.[12,52,55] Sequencing reads were mapped to the hg19 reference genome using SOLiD LifeScope V2.1 software (Life technologies, Carlsbad, CA, USA).

### Prioritization of variants

Variants were annotated using an in-house annotation pipeline, as described previously.[12,52,55] High-confidence calls (i.e. ≥10 reads, ≥5 variant reads, ≥5 unique starts (available only for substitutions) and ≥28% variant reads) were filtered for non-synonymous variants that were absent in our in-house variant database. This in-house variant database consists of variants identified by exome sequencing in 2,037 individuals from mostly Western European Ancestry (>95%) that were sequenced using SureSelect Human All Exon kit V2 and SOLiD4 sequencers (~64% of exomes), Exon kit V4 and 5500xl sequencers (~25% of exomes), or Exon kit V4 and Illumina HiSeq 2500 sequencers (~11% of exomes). All SOLiD4 and 5500xl exomes were mapped and called with LifeScope V2.1 software and the Illumina exomes were mapped with BWA and called using GATK. All exomes were sequenced at a median coverage of at least 50-fold. Next, we removed all variants present with a MAF of >0.001 in The National Heart, Lung, and Blood Institute Exome Sequencing Project database (6,503 exomes),[11] and genes that were described to be loss-of-function tolerant.[56]

Subsequently, variants that resulted in protein truncations (i.e. putative frameshifts, nonsense mutations and variants at canonical splice-sites), and non-synonymous variants with a PhyloP score ≥3.0 (missense mutations) were selected (S3 Table). The remaining list was analyzed for variants in known CRC predisposing genes and genes that predispose to other types of cancer and/or cause recessive cancer syndromes.[12,13,57]

In the next filter step, all recurrent variants and genes recurrently affected were selected. This list of variants was subsequently filtered based on the occurrence of rare potentially pathogenic variants in a whole-exome sequence data set of 164 anonymous healthy controls (Control dataset 1) that were extracted from a study reported by de Ligt et al.[55] These exomes were sequenced using a SureSelect Human All Exon kit V2 and SOLiD4 sequencers, mapped and called using SOLiD LifeScope V2.1 software, after which a filtering appoach identical to the one described for our CRC discovery cohort was applied. Analysis by a genotype-weighted metric described by Heinrich et al. [58] revealed that the exomes of the CRC patients and those from ‘Control dataset 1’ were comparable, both in terms of genetic origin and sequencing performance (S1 Fig). Significance of differences between the frequencies of variants in our CRC discovery cohort and this dataset of 164 controls were calculated using the χ^2^ goodness of fit test and a Benjamini-Hochberg (BH) correction for multiple testing was performed (corrected for 196 genes that harbored recurrent variants or were recurrently affected in our CRC discovery cohort). A *P*-value of ≤0.05 was considered significant before correction. After correction a χ^2^ *P*-value less than the BH critical value was considered significant. None of the genes were significantly enriched in our CRC discovery cohort after correcting for multiple testing. Therefore, we continued with the genes that revealed a χ^2^ goodness of fit *P*-value of ≤0.05 before multiple testing. This remaining list of variants was subjected to the following prioritization (Fig 1): (i) genes recurrently affected by protein-truncating variants solely; (ii) potential CRC driver genes (S8 Table) [24]; (iii) genes identified in transposon-based CRC susceptibility studies in mice [29,30] and involved in CRC-related KEGG pathways [pathways hsa04310 (WNT signaling), hsa04350 (TGF-beta signaling), hsa03430 (mismatch repair), hsa03410 (base excision repair), hsa03420 (nucleotide excision repair), map03450 (non-homologous end-joining), hsa03460 (Fanconi anemia), and hsa05200 (pathways in cancer)] (S9 Table); and (iv) genes identified through GWAS studies [5–7,32]. Prioritized variants were further analyzed *in silico* using software packages Align-GVGD, SIFT and Polyphen-2 integrated in the Alamut 2.0 software package (Interactive Biosoftware), and ‘HOPE’ (http://www.cmbi.ru.nl/hope) was used to assess the structural effects of missense variants at the protein level.[59] All selected variants were validated using Sanger sequencing.

### Comparisons to control populations

To investigate whether the variants we identified were truly rare, we analyzed their frequency in the dataset from the Exome Aggregation Consortium [33] and in an independent exome data set from a cohort of individuals of mostly Western-European/Dutch ancestry (Control dataset 2; *n =* 2,329) from which paired-end exome sequencing data were available with an average >90-fold coverage for our candidate genes (Agilent V4 kit; Illumina HiSeq 2500, S2 Fig). This cohort did not contain any cases with a suspected hereditary form of cancer. In addition, we assessed the frequency of potentially pathogenic variants with a MAF ≤0.001 in our candidate genes in the latter dataset. Comparisons between the Discovery cohort and ‘Control dataset 2’ were performed using the Fisher’s exact test followed by a Benjamini-Hochberg correction for the three candidate genes and for exome-wide multiple testing. A *P*-value of ≤0.05 was considered significant.

### Targeted re-sequencing

A multiplex AmpliSeq panel (Ion AmpliSeq Designer, Life technologies) was designed, targeting all coding exons of *EMR3*, *PTPN12* and *LRP6*. For library preparation, four DNA samples from the replication cohort were equimolarly pooled and used for amplification. Libaries were barcoded using Ion Xpress Barcode adapters, run on an OneTouch emulsion PCR system (Life Technologies), and sequenced using three Ion 318 chips (Life Technologies). Variant calling and annotation were performed using SeqNext (JSI Medical Systems, Kippenheim, Germany). Variants with a MAF of ≤0.001 in the EVS that resulted in protein truncations, splice-site defects, or missense mutations with a PhyloP ≥3.0 were selected and validated by Sanger sequencing in the four samples of the subsequent pool. The frequencies of variants in this CRC replication cohort and the ‘Control dataset 2’ were calculated using the Fisher’s exact test, and a Benjamini-Hochberg correction for multiple testing was performed. A *P*-value of ≤0.05 was considered significant.

### LRP6 localization and expression analyses

The human image clone IRCMp5012G1125D (Source BioScience, Nothingham, UK) was used to amplify the full-length cDNA of *LRP6*. This cDNA was subcloned into a cTAP vector in front of an in-frame C-terminal Flag-tag using the Gateway cloning system (Invitrogen, Carlsbad, CA, USA). Mutations were introduced using site-directed mutagenesis, and the open reading frames were confirmed by Sanger sequencing. For localization studies, CHO cells were grown on microscope glass slides and transfected with the wild-type or mutant LRP6 constructs using an X-tremeGENE tranfection reagent (Invitrogen). After 48 hrs, cells were fixed, incubated with a rabbit polyclonal anti-Flag antibody (Sigma Aldrich, St. Louis, USA), embedded in Vectashield with DAPI, and analyzed by fluorescence microscopy. Expression of wild-type or mutant LRP6 was compared by Western blot analyses using a rabbit polyclonal anti-Flag antibody and a goat polyclonal anti-actin antibody (Santa Cruz) and visualized using an Odyssey infrared system (Li-cor Biosciences, NE, USA).

### TOPflash reporter assay

CHO cells were transfected in 96-well plates with wild-type or mutant *LRP6* constructs together with TOP- or FOPflash reporter constructs (plasmid 12456 and 12457; Addgene, Cambridge, MA, USA) using an X-tremeGENE transfection reagent (Invitrogen).[60] The culture medium was refreshed 24 hrs post-transfection with normal or WNT3a-conditioned medium, and luciferase activity was measured after 16 hrs using a Dual-Glo Luciferase kit (Promega) and an InfiniteM200-Pro plate reader (Tecan). The expression of the reporter was normalized to co-transfected Renilla luciferase. Differences between wild-type or mutant LRP6 were assessed using a student’s t-test. A *P*-value of ≤0.05 was considered significant.

## Supporting Information

S1 Table Clinical characteristics of CRC discovery cohort. (DOCX)

S2 Table Exome performance of CRC discovery cohort. (DOCX)

S3 Table All high-confident protein truncating variants (i.e., putative frameshifts, nonsense mutations and variants at canonical splice-sites) and non-synonymous variants with a PhyloP score ≥3.0 (missense mutations) that were absent from an in-house variant database (n = 2,037 exomes) and with a MAF of ≤0.001 in NHLBI-EVS database (*n* = 6,503 exomes). N/A: not applicable.
(XLSX)

S4 Table Variants identified in known CRC predisposing genes. (DOCX)

S5 Table Variants identified in cancer (syndrome) predisposing genes. (DOCX)

S6 Table Genes with homozygous or compound heterozygous variants. (DOCX)

S7 Table Significance of differences between our CRC discovery cohort and ‘Control dataset 1’ (164 control exomes) were calculated using the χ^2^ goodness of fit test and a Benjamini-Hochberg (BH) correction for multiple testing was performed (correcting for 196 genes that harbored recurrent variants or were recurrently affected in the CRC discovery cohort). A *P*-value of ≤0.05 (indicated by an asterisk) was considered significant before correction. After correction a χ^2^ *P*-value less than the BH critical value was considered significant. None of the genes were significantly enriched in our CRC discovery cohort after correcting for multiple testing (N.S.: not significant; *: significant *P*-value of ≤0.05 before correction).
(XLSX)

S8 Table Genes that have been identified as (potential) CRC driver genes. (DOCX)

S9 Table Variants in genes previously identified in transposon-based CRC susceptibility studies and their involvement in cancer-related KEGG pathways. (DOCX)

S10 Table Enrichment analysis-based candidate genes in discovery cohort versus ‘Control cohort 2’. (DOCX)

S11 Table Variants in genes previously identified as candidate CRC susceptibility genes. (DOCX)

S1 Fig Similarities of exome samples. The similarity of the CRC cohort (*n* = 55) and the control cohort (*n* = 164) was analyzed and compared to variant sets from the 1000 genomes project using a genotype frequency weighted metric described by Heinrich et al. (2013). The results are visualized by non-metric multidimensional scaling. CRC exomes (red) and control exomes (black) cluster together, indicating similar genotyping accuracy.
(DOCX)

S2 Fig **Average coverage per exon of A) *EMR3*, B) *PTPN12* and C) *LRP6* in a control cohort of exomes of mostly Western-European ancestry (*n =* 2,329).** The average coverage is based on a representative set of 50 exomes. Error bars represent the minimal and maximal coverage per exon.
(DOCX)
