# Supplementary material for: Identification of Novel Candidate Genes for Early-Onset Colorectal Cancer Susceptibility
Source: PLoS Genet. 2016 Feb 22;12(2):e1005880. doi: 10.1371/journal.pgen.1005880 (PMC4764646; doi:10.1371/journal.pgen.1005880)
Supplement: S1 Table — (DOCX) [file pgen.1005880.s001.docx]

**S1 Table: Clinical characteristics of CRC discovery cohort.**

| Sample Name | Gender | Age of onset | Familial history for cancer^a^ | Polyps | Location | Tumor type^b^ | LN^c^ Metastasis |
| --- | --- | --- | --- | --- | --- | --- | --- |
| P001 | Male | 23 | No FHC | NO | Rectum | Adeno (NS) | NO |
| P002 | Female | 24 | 1B45; 1B/OV63-64; ?GC26; 2B60 | NO | Sigmoid | Adeno (M) | YES |
| P003 | Female | 28 | No FHC | YES | Rectum | Adeno (NS) | NO |
| P004 | Female | 28 | No FHC | YES | Sigmoid | Adeno (M) | YES |
| P005 | Male | 28 | No FHC | NO | Rectum | Signetring cell (NS) | YES |
| P006 | Female | 28 | Unknown | NO | Unknown | Unknown | NO |
| P007 | Female | 29 | 2CRC? | NO | Caecum | Squamous cell  (NS) | NO |
| P008 | Female | 29 | No FHC | YES | Unknown | Unknown | NO |
| P009 | Female | 29 | Unknown | NO | Rectum | Adeno (NS) | YES |
| P010 | Female | 30 | 2CRC80 | NO | Caecum | Adeno (NS) | YES |
| P011 | Male | 31 | No FHC | NO | Rectosigmoid | Adeno (M) | YES |
| P012 | Male | 31 | No FHC | NO | Rectum | Adeno (NS) | NO |
| P013 | Female | 32 | 2CRC71/72; 2CRC? | NO | Rectum | Intramucosal  (NS) | NO |
| P014 | Male | 32 | No FHC | YES | Unknown | Unknown | NO |
| P015 | Female | 33 | 1OV39; 2CRC56; 2CRC80 | NO | Rectosigmoid | Adeno (M) | NO |
| P016 | Female | 33 | No FHC | YES | Rectum | Adeno (M/W) | YES |
| P017 | Male | 33 | No FHC | NO | Rectum | Adeno (NS) | YES |
| P018 | Male | 33 | No FHC | NO | Sigmoid | Adeno (M) | NO |
| P019 | Male | 33 | Unknown | NO | Rectum | Adeno (NS) | NO |
| P020 | Male | 34 | 1CRC60; 2CRC70 | NO | Rectum | Adeno (M) | NO |
| P021 | Male | 34 | No FHC | NO | Appendix | Adeno (NS) | YES |
| P022 | Male | 34 | No FHC | NO | Caecum/  Ascending | Adeno (M/W) | NO |
| P023 | Female | 34 | No FHC | NO | Rectum | Adeno (P) | YES |
| P024 | Female | 34 | No FHC | NO | Rectum | Adeno (M) | YES |
| P025 | Female | 34 | Unknown | NO | Rectum | Adeno (NS) | NO |
| P026 | Female | 35 | 1CRC76 | NO | Sigmoid | Adeno (M) | NO |
| P027 | Female | 35 | No FHC | NO | Rectum | Adeno (M) | NO |
| P028 | Female | 36 | ?B35 | NO | Ascending | Unknown | NO |
| Supplementary Table S1 continued | | | | | | | |
| P029 | Female | 36 | 1CRC71 | NO | Descending | Adeno (NS) | NO |
| P030 | Female | 36 | 2CRC60 | NO | Sigmoid | Adeno (M) | YES |
| P031 | Female | 36 | 2CRC74;2CRC70 | NO | Rectum | Adeno (NS) | NO |
| P032 | Female | 36 | 3CRC45;3CRC50 | NO | Rectum | Adeno (NS) | NO |
| P033 | Male | 36 | No FHC | YES | Rectum | Adeno (W) | NO |
| P034 | Male | 37 | 1CRC63; 1CRC67 | NO | Sigmoid | Adeno (M) | NO |
| P035 | Male | 37 | 2CRC81; 2CRC75 | NO | Sigmoid | Adeno (NS) | YES |
| P036 | Female | 37 | No FHC | NO | Descending | Adeno (NS) | NO |
| P037 | Female | 37 | No FHC | NO | Descending/  Sigmoid | Adeno (NS) | NO |
| P038 | Female | 37 | No FHC | NO | Rectum | Adeno (NS) | NO |
| P039 | Female | 37 | No FHC | NO | Sigmoid | Adeno (W) | NO |
| P040 | Male | 37 | No FHC | NO | Sigmoid | Adeno (NS) | NO |
| P041 | Male | 37 | No FHC | NO | Sigmoid | Adeno (W) | NO |
| P042 | Female | 37 | No FHC | NO | Unknown | Unknown | NO |
| P043 | Female | 38 | 1CRC70;2CRC80 | NO | Rectosigmoid | Adeno (NS) | NO |
| P044 | Male | 38 | 2CRC80 | YES | Sigmoid | Adeno (M) | YES |
| P045 | Male | 38 | 2EC79 | NO | Rectum | Adeno (M) | YES |
| P046 | Female | 39 | 1SC30; 1SC>50 | NO | Ascending | Adeno (W) | YES |
| P047 | Male | 39 | 2CRC? | NO | Rectum | Adeno (NS) | NO |
| P048 | Female | 39 | 2CRC59 | NO | Unknown | Adeno (NS) | NO |
| P049 | Female | 39 | 3CRC42 | NO | Rectosigmoid | Adeno (NS) | NO |
| P050 | Male | 39 | ?CRC53 | NO | Sigmoid | Adeno (P) | YES |
| P051 | Male | 39 | No FHC | NO | Transverse | Adeno (P) | YES |
| P052 | Male | 39 | No FHC | NO | Unknown | Unknown | NO |
| P053 | Female | 40 | 1CRC79; 2CRC52; 2CRC/OV64 | NO | Transverse | Adeno (M) | NO |
| P054 | Male | 43 | No FHC | YES | Rectum | Adeno (M/W) | NO |
| P055 | Male | 45 | 1CRC60; 3CRC30 | NO | Ascending | Adeno (M) | YES |

^a^Family history for cancer (FHC): 1, 2 or 3 indicate 1^st^, 2^nd^ or 3^rd^ degree relatives; B, breast cancer; CRC, colorectal cancer; EC, endometrium cancer; GC, gastric cancer; OV, ovarian cancer; SC, skin cancer; ?, degree of relative or age unknown; age of onset is indicated after the cancer type. ^b^Tumor type and differentiation: M, moderately differentiated; W, well differentiated; P, poorly differentiated; NS, differentiation not specified. ^c^LN, Lymph node.
