# Supplementary material for: Identification of Novel Candidate Genes for Early-Onset Colorectal Cancer Susceptibility
Source: PLoS Genet. 2016 Feb 22;12(2):e1005880. doi: 10.1371/journal.pgen.1005880 (PMC4764646; doi:10.1371/journal.pgen.1005880)
Supplement: S2 Table — (DOCX) [file pgen.1005880.s002.docx]

**S2 Table: Exome performance of CRC discovery cohort.**

| Sample | Enrichment | Total bases (Gb) | Total bases on/near target (Gb) | Average Coverage  of targets | Median Coverage | % regions >= 10x | % regions >= 20x | Variants called |
| --- | --- | --- | --- | --- | --- | --- | --- | --- |
| P001 | V4-50Mb | 6.33 | 4.74 | 75.14 | 55.00 | 92.02 | 82.85 | 49929 |
| P002 | V4-50Mb | 4.36 | 3.50 | 56.97 | 45.00 | 91.22 | 80.68 | 48581 |
| P003 | V4-50Mb | 4.39 | 3.62 | 58.41 | 45.00 | 91.08 | 79.96 | 49019 |
| P004 | V2-50Mb | 7.28 | 5.72 | 97.59 | 75.00 | 87.08 | 81.83 | 43114 |
| P005 | V2-50Mb | 7.94 | 5.92 | 99.60 | 75.50 | 86.80 | 81.13 | 43786 |
| P006 | V2-50Mb | 5.84 | 4.89 | 85.07 | 68.00 | 86.11 | 80.49 | 43601 |
| P007 | V2-50Mb | 6.79 | 4.00 | 67.51 | 52.00 | 83.37 | 75.44 | 39883 |
| P008 | V2-50Mb | 8.72 | 5.36 | 87.13 | 68.00 | 84.89 | 78.93 | 37792 |
| P009 | V2-50Mb | 6.47 | 3.85 | 65.37 | 51.00 | 82.57 | 74.55 | 38656 |
| P010 | V2-50Mb | 5.18 | 4.37 | 75.85 | 60.50 | 85.00 | 78.58 | 45053 |
| P011 | V2-50Mb | 6.66 | 5.37 | 92.20 | 70.50 | 88.54 | 83.44 | 40992 |
| P012 | V2-50Mb | 6.26 | 5.21 | 89.21 | 71.00 | 86.55 | 80.94 | 44788 |
| P013 | V2-50Mb | 6.12 | 3.35 | 56.43 | 42.50 | 80.71 | 71.48 | 29040 |
| P014 | V2-50Mb | 6.54 | 5.18 | 83.75 | 65.00 | 85.79 | 79.68 | 44146 |
| P015 | V2-50Mb | 5.87 | 4.55 | 77.65 | 60.00 | 83.39 | 76.65 | 41954 |
| P016 | V2-50Mb | 5.01 | 3.93 | 63.22 | 48.00 | 83.43 | 75.25 | 40174 |
| P017 | V2-50Mb | 6.90 | 4.06 | 69.18 | 54.00 | 84.58 | 76.85 | 39804 |
| P018 | V2-50Mb | 5.91 | 3.29 | 54.93 | 43.00 | 81.94 | 72.68 | 38176 |
| P019 | V2-50Mb | 6.62 | 5.54 | 95.66 | 77.00 | 86.51 | 81.13 | 44406 |
| P020 | V2-50Mb | 5.32 | 3.30 | 57.15 | 44.00 | 83.96 | 74.91 | 38551 |
| P021 | V2-50Mb | 7.06 | 4.24 | 70.27 | 53.00 | 84.91 | 77.66 | 40331 |
| P022 | V2-50Mb | 6.59 | 5.12 | 87.23 | 67.00 | 85.18 | 79.00 | 42542 |
| P023 | V4-50Mb | 5.79 | 4.52 | 76.15 | 60.00 | 92.89 | 85.59 | 47370 |
| P024 | V2-50Mb | 4.37 | 3.52 | 56.12 | 44.00 | 83.06 | 74.98 | 43396 |
| P025 | V2-50Mb | 9.49 | 6.00 | 101.65 | 77.00 | 87.63 | 82.61 | 39429 |
| P026 | V2-50Mb | N/A | 4.46 | 74.74 | 57.50 | 85.02 | 78.49 | 38803 |
| P027 | V2-50Mb | 6.25 | 4.85 | 81.59 | 61.00 | 83.77 | 76.98 | 42404 |
| P028 | V4-50Mb | 4.60 | 3.76 | 60.80 | 48.00 | 92.05 | 82.15 | 49423 |
| P029 | V2-50Mb | 4.34 | 3.40 | 57.79 | 45.00 | 82.50 | 74.34 | 39716 |
| P030 | V2-50Mb | 6.85 | 5.78 | 101.89 | 82.00 | 87.99 | 83.28 | 44805 |
| P031 | V4-50Mb | 5.25 | 4.25 | 69.45 | 54.00 | 92.47 | 84.04 | 49502 |
| P032 | V4-50Mb | 6.24 | 5.06 | 81.03 | 63.00 | 93.99 | 87.15 | 49800 |
| Supplementary Table S2 continued | | | | | | | | |
| P033 | V1-30Mb | N/A | 2,78 | 46.68 | 48.50 | 73.47 | 65.36 | 36324 |
| P034 | V2-50Mb | 6.20 | 4.57 | 76.86 | 59.50 | 83.28 | 76.65 | 41507 |
| P035 | V2-50Mb | 6.98 | 5.74 | 98.44 | 78.00 | 87.47 | 82.36 | 45328 |
| P036 | V2-50Mb | 6.66 | 5.48 | 93.47 | 74.00 | 87.16 | 82.02 | 45973 |
| P037 | V2-50Mb | 8.86 | 6.76 | 114.35 | 87.00 | 86.94 | 81.87 | 43724 |
| P038 | V2-50Mb | 8.53 | 7.16 | 127.29 | 102.0 | 89.46 | 85.59 | 45725 |
| P039 | V2-50Mb | 6.90 | 5.37 | 91.72 | 72.00 | 86.39 | 80.92 | 42867 |
| P040 | V2-50Mb | 6.29 | 5.14 | 84.90 | 67.00 | 87.11 | 81.51 | 46267 |
| P041 | V2-50Mb | 7.18 | 6.03 | 105.27 | 84.00 | 87.58 | 82.60 | 45089 |
| P042 | V2-50Mb | 5.37 | 4.15 | 70.45 | 54.50 | 83.28 | 76.03 | 40257 |
| P043 | V2-50Mb | 6.15 | 4.94 | 78.50 | 62.00 | 85.73 | 79.92 | 44700 |
| P044 | V2-50Mb | 6.39 | 5.07 | 85.36 | 64.00 | 85.44 | 79.14 | 41855 |
| P045 | V2-50Mb | 6.13 | 4.73 | 81.03 | 62.00 | 83.48 | 76.65 | 41743 |
| P046 | V2-50Mb | 5.48 | 4.23 | 74.32 | 57.00 | 84.70 | 77.65 | 41289 |
| P047 | V4-50Mb | 4.66 | 3.70 | 57.02 | 41.00 | 89.08 | 75.98 | 47408 |
| P048 | V2-50Mb | 5.72 | 4.45 | 70.99 | 56.00 | 84.85 | 78.39 | 45070 |
| P049 | V2-50Mb | 6.71 | 5.40 | 86.62 | 68.00 | 86.89 | 81.73 | 45210 |
| P050 | V2-50Mb | 4.01 | 3.25 | 53.35 | 42.00 | 83.37 | 74.55 | 43623 |
| P051 | V2-50Mb | 4.38 | 3.26 | 53.54 | 42.00 | 80.43 | 71.11 | 40509 |
| P052 | V2-50Mb | 5.78 | 4.14 | 67.90 | 53.00 | 82.14 | 75.06 | 41337 |
| P053 | V4-50Mb | 3.83 | 3.03 | 47.44 | 35.00 | 87.42 | 72.12 | 46226 |
| P054 | V2-50Mb | 5.92 | 4.19 | 66.33 | 51.00 | 83.39 | 76.05 | 41973 |
| P055 | V4-50Mb | 4.29 | 3.46 | 56.71 | 44.00 | 90.56 | 79.20 | 48893 |

Abbreviations: V1, Version 1; V2, Version 2; V4, Version 4 Agilent exome enrichment kits; N/A, not available.
