# Supplementary material for: Identification of Novel Candidate Genes for Early-Onset Colorectal Cancer Susceptibility
Source: PLoS Genet. 2016 Feb 22;12(2):e1005880. doi: 10.1371/journal.pgen.1005880 (PMC4764646; doi:10.1371/journal.pgen.1005880)
Supplement: S4 Table — (DOCX) [file pgen.1005880.s004.docx]

**S4 Table: Variants identified in known CRC predisposing genes.^a^**

| Sample | Gene | Chr | Start | End | Ref | Var | PhyloP | Refseq. Accession | Protein effect | dbSNP | EVS MAF | Gene type |
| --- | --- | --- | --- | --- | --- | --- | --- | --- | --- | --- | --- | --- |
| P022 | *MSH2* | 2 | 47635601 | 47635603 | TCT | - | N/A | NM_000251 | p.D91del | - | - | CRC |
| P025 | *MSH6* | 2 | 48033981 | 48033981 | - | TTGA | N/A | NM_000179 | p.T1355Tfs* | - | - | CRC |
|  |  |  | 48032098 | 48032098 | A | T | 4.788 | NM_000179 | p.E1163V | rs63750252 | - | CRC |
| P045 | *MSH6* | 2 | 48030685 | 48030685 | C | T | 4.398 | NM_000179 | p.T1100M | rs63750442 | - | CRC |
| P017 | *PMS2* | 7 | 6043689 | 6043689 | T | G | 4.769 | NM_000535 | p.D55A | - | - | CRC |

Abbreviations: Chr, chromosome; Ref, reference allele; Var, variant allele; EVS, exome variant server; MAF, minor allele frequency.

^a^All variants were validated with Sanger sequencing.
