# Supplementary material for: Identification of Novel Candidate Genes for Early-Onset Colorectal Cancer Susceptibility
Source: PLoS Genet. 2016 Feb 22;12(2):e1005880. doi: 10.1371/journal.pgen.1005880 (PMC4764646; doi:10.1371/journal.pgen.1005880)
Supplement: S5 Table — (DOCX) [file pgen.1005880.s005.docx]

**S5 Table: Variants identified in cancer (syndrome) predisposing genes.^a^**

| Sample | Gene | Chr | Start | End | Ref | Var | PhyloP | Refseq. Accession | Protein effect | dbSNP | EVS MAF | Gene type |
| --- | --- | --- | --- | --- | --- | --- | --- | --- | --- | --- | --- | --- |
| P008 | *BLM* | 15 | 91346951 | 91346951 | G | T | 5.193 | NM_000057 | Splicing | rs148969222 | 0.000077 | Cancer |
| P034 | *BLM* | 15 | 91328183 | 91328183 | C | T | 0.363 | NM_000057 | p.R899* | - | - | Cancer |
| P001 | *ATM^b^* | 11 | 108216611 | 108216611 | C | T | 6.107 | NM_000051 | p.R2854C | - | - | Cancer |
| P011 | *ATM^b^* | 11 | 108159711 | 108159711 | G | C | 5.596 | NM_000051 | p.D1373N | - | - | Cancer |
| P005 | *BRIP1* | 17 | 59934544 | 59934544 | G | A | 3.159 | NM_032043 | p.S85L | - | - | Cancer |
| P011 | *EGFR* | 7 | 55259460 | 55259460 | G | A | 5.927 | NM_005228 | p.A840T | rs143884981 | 0.000077 | Cancer |
| P040 | *ERCC3* | 2 | 128046346 | 128046346 | A | G | 5.030 | NM_000122 | p.I306T | - | - | Cancer |
| P039 | *WRN* | 8 | 30989910 | 30989910 | C | G | 1.597 | NM_000553 | p.S952* | - | - | Cancer |

Abbreviations: Chr, chromosome; Ref, reference allele; Var, variant allele; EVS, exome variant server; MAF, minor allele frequency.

^a^All variants were validated with Sanger sequencing.

^b^A third missense variant in *ATM* was not validated by Sanger sequencing.
