# Supplementary material for: Identification of Novel Candidate Genes for Early-Onset Colorectal Cancer Susceptibility
Source: PLoS Genet. 2016 Feb 22;12(2):e1005880. doi: 10.1371/journal.pgen.1005880 (PMC4764646; doi:10.1371/journal.pgen.1005880)
Supplement: S6 Table — (DOCX) [file pgen.1005880.s006.docx]

**S6 Table: Genes with homozygous or compound heterozygous variants.^a^**

| **Sample** | **Gene** | **Chr** | **Start** | **End** | **Ref** | **Var** | **%**  **Variation** | **PhyloP** | **Refseq. Accession** | **Protein effect** | **dbSNP** | **Recessive family** | **Comment** |
| --- | --- | --- | --- | --- | --- | --- | --- | --- | --- | --- | --- | --- | --- |
| **P02** | *ZPLD1* | 3 | 102171825 | 102171825 | A | C | 100.0 | 3.353 | NM_175056 | p.T73P | - | No | Gene function unknown |
| **P025** | *MSH6* | 2 | 48033981 | 48033981 | - | TTGA | 30.9 | N/A | NM_000179 | p.T1355Tfs* | - | Unknown | Tumor does not show MSI |
|  |  |  | 48032098 | 48032098 | A | T | 31.8 | 4.788 |  | p.E1163V | rs63750252 |  |  |
| **P027** | *ESPN* | 1 | 6505934 | 6505934 | A | G | 100.0 | 4.00 | NM_031475 | p.D468G | rs145306517 | Potentially | Associated with other phenotype (MIM: 609006) |
| **P028** | *FRRS1L* | 9 | 111899877 | 111899877 | G | A | 95.2 | 5.742 | NM_014334 | p.P298L | - | Potentially | Gene function unknown |
| **P31** | *DYNC2H1* | 11 | 103024105 | 103024105 | G | A | 50.0 | 3.696 | NM_001080463 | p.L3633F | - | Potentially | Associated with other phenotype (MIM: 613091) |
|  |  |  | 103339392 | 103339392 | G | A | 42.3 | 3.696 |  | p.R1057F | rs191381310 |  |  |
| **P35** | *ATP13A1* | 19 | 19767843 | 19767843 | G | C | 100.0 | 3.388 | NM_020410 | p.S289W | - | Potentially | Gene function unknown |
| **P41** | *NUFIP2* | 17 | 27613578 | 27613578 | C | T | 42.3 | 4.352 | NM_020772 | p.M478I | - | Potentially | Gene function unknown |
|  |  |  | 27614371 | 27614371 | G | T | 36.8 | 6 |  | p.S214Y | - |  |  |
| **P45** | *TTN* | 2 | 179406086 | 179406086 | C | T | 38.4 | 6.197 | NM_133378 | p.R30005H | - | Potentially | Associated with other phenotypes (MIM:  188840) |
|  |  |  | 179397346 | 179397346 | G | C | 33.6 | 4.539 |  | p.P32098A | - |  |  |

Abbreviations: Chr, chromosome; Ref, reference allele; Var, variant allele; ND, Not done.

^a^All variants were validated with Sanger sequencing.
