# Supplementary material for: Identification of Novel Candidate Genes for Early-Onset Colorectal Cancer Susceptibility
Source: PLoS Genet. 2016 Feb 22;12(2):e1005880. doi: 10.1371/journal.pgen.1005880 (PMC4764646; doi:10.1371/journal.pgen.1005880)
Supplement: S8 Table — (DOCX) [file pgen.1005880.s008.docx]

**S8 Table: Genes that have been identified as (potential) CRC driver genes.**

| *ACVR1B* | *CDC27* | *FZD3* | *MIER3* | *PIK3CA* | *TCERG1* |
| --- | --- | --- | --- | --- | --- |
| *ACVR2A* | *CTNNB1* | *GPC6* | *MSH3* | *PTPN12* | *TCF7L2* |
| *APC* | *EDNRB* | *KIAA1804* | *MSH6* | *SMAD2* | *TGFBR2* |
| *BRAF* | *FAM123B* | *KRAS* | *MYO1B* | *SMAD4* | *TP53* |
| *CASP8* | *FBXW7* | *MAP7* | *NRAS* | *SOX9* | *TTN* |
