# Supplementary material for: Identification of Novel Candidate Genes for Early-Onset Colorectal Cancer Susceptibility
Source: PLoS Genet. 2016 Feb 22;12(2):e1005880. doi: 10.1371/journal.pgen.1005880 (PMC4764646; doi:10.1371/journal.pgen.1005880)
Supplement: S9 Table — (DOCX) [file pgen.1005880.s009.docx]

**S9 Table: Variants in genes previously identified in transposon-based CRC susceptibility studies and their involvement in cancer-related KEGG pathways.**

| Sample | Gene | Chr | Start | End | Ref | Var | %  Variation | PhyloP | Refseq. Accession | Protein effect | dbSNP | KEGG CRC  Pathway | Sanger |
| --- | --- | --- | --- | --- | --- | --- | --- | --- | --- | --- | --- | --- | --- |
| P001 | *LRP6* | 12 | 12312812 | 12312812 | T | C | 40.8 | 5.097 | NM_002336 | p.N789S | - | WNT | Confirmed |
| P002 | *LRP6* | 12 | 12339985 | 12339985 | C | A | 51.7 | 6.244 | NM_002336 | p.W239L | - | WNT | Confirmed |
| P008 | *LRP6* | 12 | 12311955 | 12311955 | T | C | 29.6 | 5.13 | NM_002336 | p.T867A | rs141458215 | WNT | Confirmed |
| P006 | *PTPRD* | 9 | 8484257 | 8484257 | G | C | 36.4 | 5.854 | NM_002839 | p.A1092G | - | - | ND |
| P007 | *LRBA* | 4 | 151788945 | 151788945 | C | T | 31.6 | 6.198 | NM_001199282 | p.D882N | rs145086254 | - | ND |
| P011 | *KCTD20* | 6 | 36446911 | 36446911 | G | A | 33.3 | 5.968 | NM_173562 | p.G150R | - | - | ND |
| P013 | *HUWE1* | X | 53595706 | 53595706 | T | C | 51.6 | 4.527 | NM_031407 | p.K2218R | - | - | ND |
| P014 | *CFTR* | 7 | 117232473 | 117232473 | G | A | 37.7 | 4.013 | NM_000492 | p.R751H | - | - | ND |
| P026 | *UBR4* | 1 | 19427040 | 19427040 | C | G | 33.8 | 5.389 | NM_020765 | p.V4351L | - | - | ND |
| P032 | *CFTR* | 7 | 117171011 | 117171011 | C | T | 28.2 | 4.467 | NM_000492 | p.P111L | rs140502196 | - | ND |
| P033 | *HUWE1* | X | 53565329 | 53565329 | T | G | 100.0 | 3.013 | NM_031407 | p.I3989L | - | - | Confirmed |
|  | *UBR4* | 1 | 19443890 | 19443890 | T | C | 41.1 | 4.43 | NM_020765 | p.I3550V | - | - | ND |
| P035 | *BIRC6* | 2 | 32832646 | 32832646 | G | A | 41.1 | 6.099 | NM_016252 | p.R4732Q | - | - | ND |
| P044 | *LRBA* | 4 | 151836786 | 151836786 | T | C | 39.6 | 5.06 | NM_001199282 | p.I330V | - | - | ND |
| P046 | *KCTD20* | 6 | 36449446 | 36449446 | C | T | 71.9 | 2.573 | NM_173562 | p.R256X | - | - | Confirmed |
| P050 | *PTPRD* | 9 | 8375990 | 8375990 | C | T | 46.8 | 6.189 | NM_002839 | p.R1536H | rs142960593 | - | ND |
|  | *UBR4* | 1 | 19420561 | 19420561 | G | A | 40.7 | 5.528 | NM_020765 | p.R4607C | - | - | ND |
| P052 | *BIRC6* | 2 | 32820146 | 32820146 | A | G | 41.9 | 3.442 | NM_016252 | p.L4516R | - | - | ND |

Abbreviations: Chr, chromosome; Ref, reference allele; Var, variant allele; ND, Not done.
