# Supplementary material for: Identification of Novel Candidate Genes for Early-Onset Colorectal Cancer Susceptibility
Source: PLoS Genet. 2016 Feb 22;12(2):e1005880. doi: 10.1371/journal.pgen.1005880 (PMC4764646; doi:10.1371/journal.pgen.1005880)
Supplement: S10 Table — (DOCX) [file pgen.1005880.s010.docx]

**S10 Table:** Enrichment analysis-based candidate genes in discovery cohort versus ‘Control cohort 2’.

| Gene | Number of variants | | | Uncorrected^c^ | Three gene correction^d^ | Exome-wide correction^e^ | Replication cohort (*n*=174) |
| --- | --- | --- | --- | --- | --- | --- | --- |
|  | **Discovery cohort (*n*=55)** | | **Control cohort 2 (*n*=2,329)** |  |  |  |  |
| *EMR3* | 2 | 12^a^ | | OR: 7.16  CI: 0.769-32.733  *P* = 0.04006 | *P* = 0.040060 | *P* = 1 | 0 |
| *PTPN12* | 3 | 11^b^ | | OR: 11.82  CI: 2.088-45.615  *P* = 0.003613 | *P* = 0.026100 | *P* = 1 | 1^f^ |
| *LRP6* | 3 | 18^b^ | | OR: 7.218  CI: 1.342-25.278  *P* = 0.0117 | *P* = 0.010839 | *P* = 1 | 0 |

^a^Total number of loss-of-function variants (e.g. nonsense, frameshift or splice site variants) with a MAF of ≤0.001 in NHLBI-EVS database in ‘Control cohort 2’. ^b^Total number of highly conserved missense variants (PhyloP ≥3.0) with a MAF of ≤0.001 in NHLBI-EVS database in ‘Control cohort 2’. ^c^Fisher’s exact test between Discovery cohort and ‘Control cohort 2’ not corrected for multiple testing. *^d^P*-values Fisher’s exact test between Discovery cohort and ‘Control cohort 2’ corrected for three genes. *^e^P*-values Fisher’s exact test between Discovery cohort and ‘Control cohort 2’ corrected for (exome-wide) multiple testing. ^f^Fisher’s exact test between Replication cohort and ‘Control cohort 2’ was not significant. NHLBI-EVS: Exome variant server.
