# Supplementary material for: Identification of Novel Candidate Genes for Early-Onset Colorectal Cancer Susceptibility
Source: PLoS Genet. 2016 Feb 22;12(2):e1005880. doi: 10.1371/journal.pgen.1005880 (PMC4764646; doi:10.1371/journal.pgen.1005880)
Supplement: S11 Table — (DOCX) [file pgen.1005880.s011.docx]

**S11 Table: Variants in genes previously identified as candidate CRC susceptibility genes.**

| Sample  Name | Gene | Chr | Start | End | Ref | Var | %  Variation | PhyloP | Refseq. Accession | Protein effect | dbSNP | Sanger |
| --- | --- | --- | --- | --- | --- | --- | --- | --- | --- | --- | --- | --- |
| P005 | *BRIP1* | 17 | 59934544 | 59934544 | G | A | 41.3 | 3.159 | NM_032043 | p.S85L | - | Confirmed |
| P17 | *SYNE1* | 6 | 152443612 | 152443612 | G | T | 47.5 | 3.252 | NM_182961 | p.H8785N | - | ND |
| P023 | *SMARCA4* | 19 | 11169019 | 11169019 | G | A | 54.3 | 5.365 | NM_001128848 | p.V1471M | - | ND |
|  | *TWSG1* | 18 | 9360040 | 9360040 | C | A | 81.8 | 3.834 | NM_020648 | p.A65D | rs145253009 | ND |
| P043 | *UACA* | 15 | 70952527 | 70952527 | A | C | 32.0 | 4.672 | NM_018003 | p.I1381S | - | ND |

Abbreviations: Chr, chromosome; Ref, reference allele; Var, variant allele; ND, Not done.
