# Supplementary material for: Identification of Novel Candidate Genes for Early-Onset Colorectal Cancer Susceptibility
Source: PLoS Genet. 2016 Feb 22;12(2):e1005880. doi: 10.1371/journal.pgen.1005880 (PMC4764646; doi:10.1371/journal.pgen.1005880)
Supplement: S1 Fig — The similarity of the CRC cohort (n = 55) and the control cohort (n = 164) was analyzed and compared to variant sets from the 1000 genomes project using a genotype frequency weighted metric described by Heinrich et al. (2013). The results are visualized by non-metric multidimensional scaling. CRC exomes (red) and control exomes (black) cluster together, indicating similar genotyping accuracy. (DOCX) [file pgen.1005880.s012.docx]

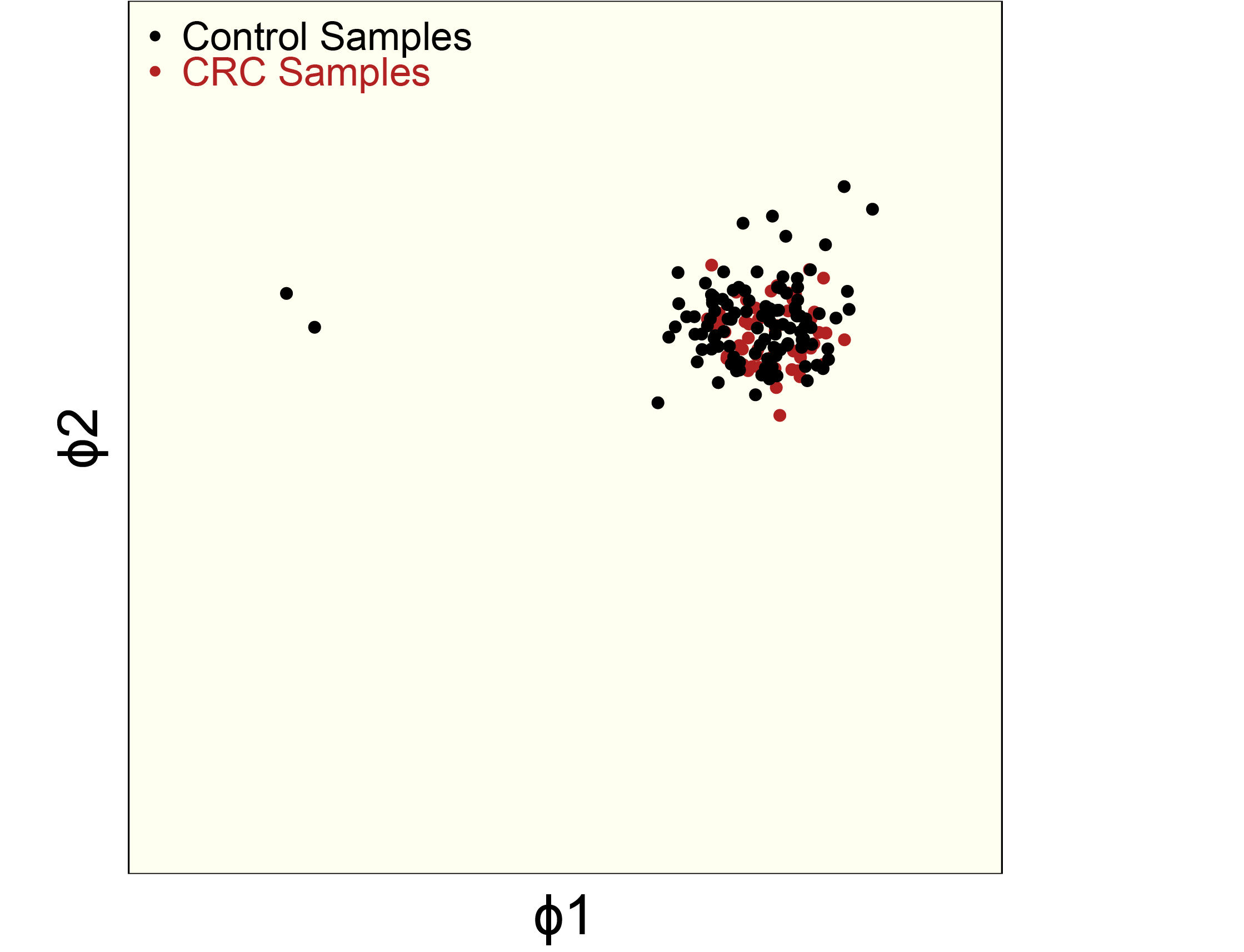


**S1 Figure:** Similarities of exome samples. The similarity of the CRC cohort (*n*=55) and the control cohort (*n*=164) were analysed and compared to variant sets from the 1000 genomes project using a genotype frequency weighted metric described by Heinrich et al [6]. The results are visualised by non-metric multidimensional scaling. CRC exomes (red) and control exomes (black) cluster together, indicating similar genotyping accuracy.
