# Supplementary material for: Identification of Novel Candidate Genes for Early-Onset Colorectal Cancer Susceptibility
Source: PLoS Genet. 2016 Feb 22;12(2):e1005880. doi: 10.1371/journal.pgen.1005880 (PMC4764646; doi:10.1371/journal.pgen.1005880)
Supplement: S2 Fig — Average coverage per exon of A) EMR3, B) PTPN12 and C) LRP6 in a control cohort of exomes of mostly Western-European ancestry (n = 2,329). The average coverage is based on a representative set of 50 exomes. Error bars represent the minimal and maximal coverage per exon. (DOCX) [file pgen.1005880.s013.docx]

**
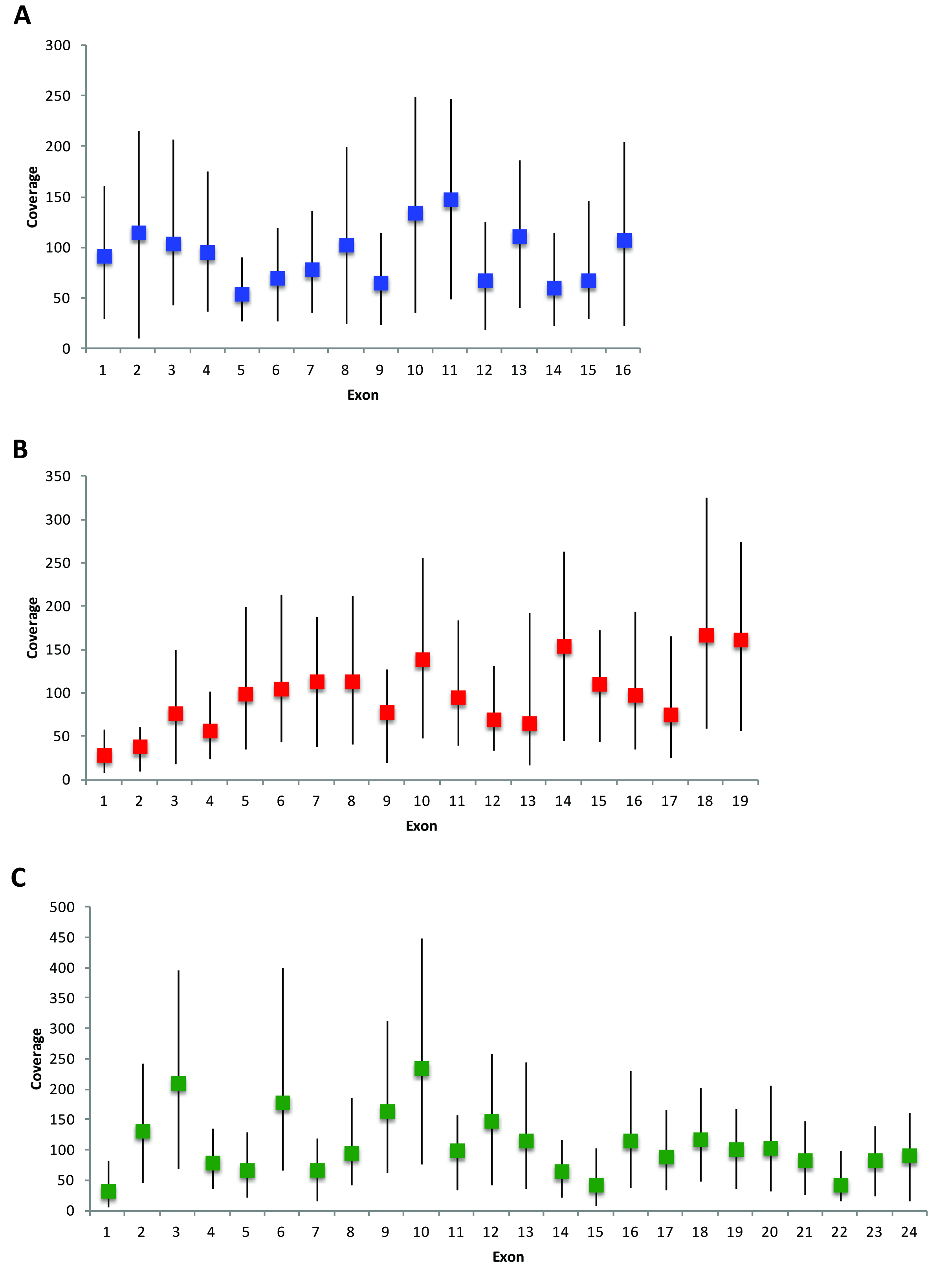
**

**S2 Figure:** Average coverage per exon of A) *EMR3,* B) *PTPN12* and C) *LRP6* in a control cohort of exomes of mostly Western-European Ancestry (*n=*2,329). Average coverage per exon is based on a representative set of 50 exomes extracted from the total dataset. Error bars represent the minimal and maximal coverage per exon.
